# Hypnagogia, psychedelics, and sensory deprivation: the mythic structure of dream-like experiences

**DOI:** 10.3389/fpsyg.2025.1498677

**Published:** 2025-05-09

**Authors:** Andreas Huber, Anette Kjellgren, Torsten Passie

**Affiliations:** ^1^Schwerelos Sensory Deprivation Laboratory, Vienna, Austria; ^2^Department of Psychology, Karlstad University, Karlstad, Sweden; ^3^Hannover Medical School, Hannover, Germany; ^4^Senckenberg Institute for History and Ethics in Medicine, Goethe University, Frankfurt am Main, Germany

**Keywords:** dreaming, dream-like experience, hypnagogia, psychedelics, sensory deprivation, flotation-REST, ontology, mythic cognition

## Abstract

**Introduction:**

Dream-like and psychedelic experiences often display internally illogical structures. Recent theories propose that these experiences function as “spontaneous offline simulations” related to specific brain processes. This study investigates whether such perceived illogicality reflects a distinct, premodern mode of cognition—commonly referred to as “mythic” cognition—rather than a cognitive deficit.

**Methods:**

Thirty-one participants underwent four 90-minute flotation REST (Restricted Environmental Stimulation Technique) sessions designed to induce altered, dream-like states. After each session, participants completed the Phenomenology of Consciousness Inventory (PCI) and additional questions targeting features associated with mythic cognition.

**Results:**

Participants showed significant phenomenological shifts toward experiences characteristic of mythic cognition. Specifically, their altered states during flotation exhibited ontological parallels with mythic conceptions of space, time, and substance.

**Discussion:**

The findings support the hypothesis that the perceived illogicality in altered states arises from a distinct cognitive framework rather than from deficits.

## Introduction

Altered states of consciousness, characterized by dream-like experiences, represent an intriguing facet of human perception. These states can be induced by different methods such as pharmacological agents (e.g., LSD-like hallucinogens) or sensory deprivation (Sanz et al., 2018; Bood, 2007; Leuner, 1962). This study focuses on dream-like and hypnagogic states, which are characterized by enhanced imagery. These states share features such as vivid sensory content, detachment from external stimuli, and varying degrees of voluntary control over immersion and imagery (cf. Vaitl et al., 2005; Mavromatis, 1987; Casey, 1976).

Sensory deprivation, such as in floating tanks, allows individuals to experience immersive, surreal states of consciousness, characterized by unusual and often bizarre dream-like experiences. While early studies (e.g., Lilly, 1977) focused on hallucinatory phenomena, more recent work has demonstrated that sensory deprivation can activate narrative and symbolic processes deeply rooted in the human psyche (Feinstein et al., 2018). An extreme example of how reduced sensory-motor feedback generates dream-like experiences is oneiroid syndrome, where individuals perceive themselves as awake but cannot distinguish these experiences from reality (Schmidt-Degenhard, 1992; Mayer-Gross, 1924). This condition can occur in cases of complete paralysis, such as in polyradiculitis (Guillain-Barré Syndrome), leading to the perception of a coherent ‘other world’ that feels entirely real. Tart (1975) describes such states as characterized by reduced exteroception and increased focus on internal content, similar to hypnagogic and dream-like imagery (Ghibellini and Meier, 2023; Schacter, 1976). Beyond physical sensory deprivation, altered states akin to dream-like experiences can also arise through pharmacological interventions. Psychedelic substances, for example, are known to disrupt networks like the default mode network, dissolving cognitive and perceptual boundaries (Carhart-Harris et al., 2014). These experiences are often perceived as mystical or archetypal, potentially reflecting a more associative and less analytically constrained cognitive mode (Griffiths et al., 2018; Luke, 2011; Masters and Houston, 1966).

These states display a certain logical incoherence, which remains complex to understand. This deviation from normal waking logic has led to various psychophysiological interpretations. For example, Jung (1995) emphasized their symbolic dimensions, while Leuner (1962) linked them to emotional tensions. However, contemporary cognitive theories suggest that dream-like states serve to process and organize daily experiences, creating illogical connections as the brain consolidates memories (Windt, 2015; Born and Wilhelm, 2012). Recent research suggests that illogical experiences, such as dreams, are not separate from waking consciousness but exist along a continuous spectrum of cognitive states, challenging the traditional binary distinction between logical waking and illogical dream states (Christoff et al., 2016; Fox et al., 2013). While older theories attributed cognitive deficits to this incoherence in dreams (Domhoff, 2019), newer perspectives, such as Ruby and Blochet (2019), propose that dreams are spontaneous mental simulations, similar to daydreaming. Revonsuo (2000) posits that dreams serve an evolutionary function by simulating reality, preparing individuals for real-life challenges and enhancing survival mechanisms. His threat simulation theory suggests that dreaming evolved primarily to rehearse responses to dangerous situations, such as predator attacks, thereby increasing survival rates. However, this perspective has been criticized for being too narrowly focused on danger.

Germain et al. (2004) argue that dreams often involve complex social interactions that are not directly related to immediate threats, highlighting the broader functions of dreaming. In a similar vein, Nielsen and Germain (2000) propose that dreams play a wider role in processing emotional experiences, including the integration of traumatic events. They suggest that dreams help reinforce attachment relationships, which were just as crucial for survival as escaping predators. By repeatedly exposing individuals to social and narrative scenarios, dreams may have aided early humans in developing stable group structures and fostering collective problem-solving.

From a broader perspective, dreams serve as “polyvalent cognitive and socio-emotional functions essential to the ongoing evolution of our species” (Nielsen and Germain, 2000), with self-imagery facilitating identity formation and spatial imagery supporting navigation and orientation. Building on this idea, Revonsuo et al. (2015) later expanded his threat simulation theory to encompass not only the simulation of dangers but also social and cognitive challenges. Similarly, Hobson and Friston (2012) suggest that dreams help reduce the complexity of Bayesian models accumulated during waking consciousness, maintaining cognitive coherence and facilitating adaptation to new information. Extending this idea, Kirberg (2022) argues that the seemingly bizarre logic of dream-like states reflects cognitive processes that operate continuously across both sleep and wakefulness.

Current psychophysiological approaches often analyze dream-like experiences by comparing them to normal waking cognition, with the waking state traditionally serving as the benchmark for logical coherence. This framework typically leads to the conclusion that dream-like states are characterized by cognitive deficits or illogical thinking, as they deviate from the structured logic of waking consciousness.

However, we propose that this perspective may be limited by the assumption that modern waking consciousness—characterized by rational and linear thinking—is the optimal or “standard” model of cognition. Our hypothesis suggests that dream-like experiences are not inherently deficient in cognitive value but instead build upon an entirely different ontological basis, one that enables symbolic, narrative, and meaning-making dynamics often found in ‘premodern’ mythic thinking.

**Figure 1:**
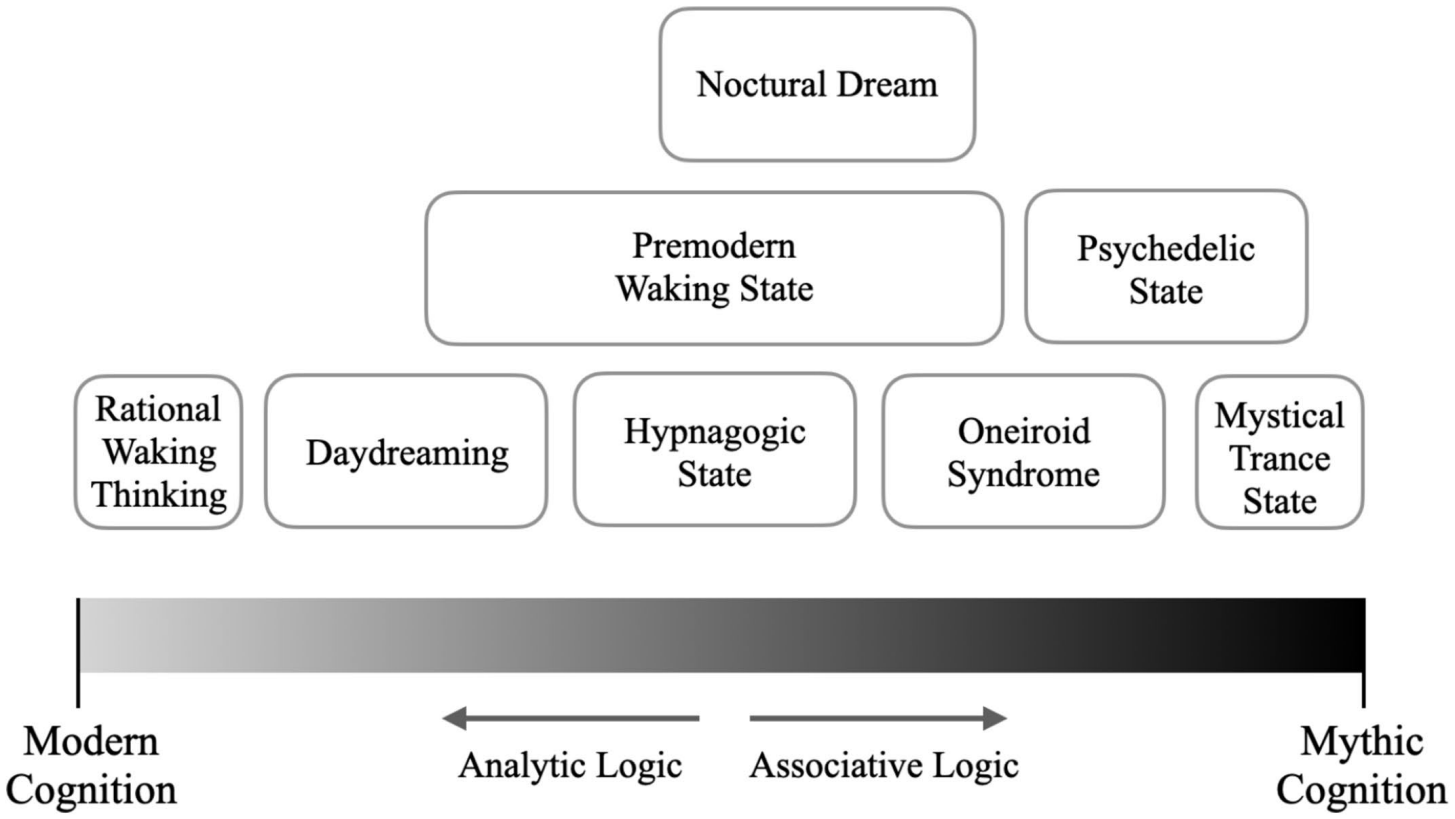
Visualization of the hypothetical cognitive continuum with a tentative mapping of different states of consciousness.

To test this hypothesis, we investigated how participants in sensory deprivation tanks—environments known to induce dream-like and hypnagogic states—perceived their experiences. Participants evaluated both their normal waking state and the dream-like experience in terms of how closely each state aligned with modern or mythic cognitive features. This approach aims to critically assess whether modern waking consciousness is indeed the most suitable model for understanding dream-like experiences or if they should be framed within a continuum ranging from modern to mythic cognition. The visual representation of this hypothetical continuum (see Figure 1) facilitates the understanding and positioning of different states of consciousness within it.

A central assumption of this study is that dream-like states are characterized by a cognitive logic more aligned with pre-modern thought patterns than modern rational paradigms. To test this hypothesis, flotation REST experiments were employed, as they are known to facilitate access to such states. To operationalize the concept of mythical cognition, ontological definitions of space, time, and substance were adapted and translated into specific, lay-accessible criteria. These criteria enabled a systematic evaluation of participants’ subjective experiences and provided a robust framework for analyzing the cognitive shifts induced by flotation REST.

## Methods

### Study design

A within-subject design was employed for this exploratory study. Sample size estimates were based on previous studies utilizing *Flotation-REST* (Bood et al., 2009; Bood et al., 2007; Kjellgren, 2003). The study took place at the *Schwerelos Sensory Deprivation Laboratory* in Vienna, Austria. The research protocol received ethical approval from the Medical University of Vienna’s ethics committee (EC No. 1714/2014). The study was performed in accord to relevant guidelines and regulations. Participants were unaware of the study’s hypotheses and underwent a 3-week program consisting of four 90-min *Flotation-REST* sessions. To explore the changes in the participants’ state of consciousness, the *Phenomenology of Consciousness Inventory questionnaire* (Pekala, 1991; see *PCI*) was administered. The participants assessed the structural logic of the dream-like experiences induced by flotation-REST using Kurt Hübner’s ontological definitions (1985; see *DMO*). Both instruments were administered before the first flotation-REST session (baseline). The first Flotation-REST session was not studied as it was provided to familiarize participants with the sensory deprivation environment, while after sessions 2, 3, and 4, the PCI was administered to evaluate the state of consciousness. The baseline measurement captured participants’ everyday waking experience in the last 1–2 h prior to their arrival at the laboratory, while the measurement immediately after each flotation-REST session aimed to reflect their subjective experience during the 90-min period of sensory isolation. After session 4, the Hübner’s definitions were administered. The results were intended to facilitate a comparison between dream-like experiences and its distinct experiential structure with the participants’ everyday waking state of consciousness.

### Participants

Participants were recruited for the study by advertisements and a dedicated website. Individuals between the ages of 18 and 65 years were screened. Inclusion criteria included no severe psychopathology and physical healthiness. Exclusion criteria encompassed (a) pregnancy, (b) pacemaker implantation, (c) open wounds or skin diseases, (d) epilepsy, (e) history of psychosis, (f) post-traumatic stress disorder, and (g) substance abuse. Subjects were screened using the *Mini International Neuropsychiatric Interview - Version 5.0* to exclude individuals exhibiting psychotic symptoms.

### Restricted environmental stimulation technique (flotation-REST)

Flotation-REST is a specialized technique used to study individuals under conditions of minimal sensory stimulation. To enhance sensory deprivation (SD), Lilly and Shurley introduced the “isolation tank” in the late 1950s. In this bathtub-like environment, subjects float in a warm saltwater solution, significantly reducing sensory input. Research has shown that a high degree of SD typically induces dream-like experiences in most subjects (Bonk, 2018; Kjellgren et al., 2008; Norlander et al., 2001; Freedman et al., 1962).

For this study, we utilized an advanced commercial isolation tank provided by RestingWell Sweden (2024). The tank, measuring 260 × 165 × 125 cm, created a comfortable, bath-like environment, filled with water to a depth of 30 cm and saturated with Epsom salt (magnesium sulfate; 1.3 g/cm^3^) to enable effortless flotation. The water temperature was maintained at 35°C. To ensure a constant supply of preheated fresh air, the tank was equipped with a noiseless ventilation system. It provided complete darkness and minimal acoustic stimulation (with earplugs also provided). The tank was easily accessible, allowing subjects to exit comfortably, and an “alarm button” inside the tank enabled them to alert laboratory staff if needed. The room housing the tank was equipped with shower and toilet facilities, and subjects were instructed to shower before and after each Flotation-REST session.

### Phenomenology of consciousness inventory (PCI)

The PCI (Phenomenology of Consciousness Inventory) is a validated questionnaire designed for retrospective self-assessment of states of consciousness. It quantifies subjective experiences based on pattern parameters, as proposed by Tart (1975), and intensity parameters, as proposed by Singer (1977). Affective qualities within the questionnaire are categorized according to Plutchik’s (1980) four primary emotional qualities: anger/rage, fear, sadness, and joy.

The PCI questionnaire consists of 53 items, each featuring two statements rated on a Likert scale from 0 to 7, designed to measure 12 dimensions of consciousness. Five of these dimensions are further divided into two or more subdimensions, resulting in a total of 14 subdimensions. To ensure intratest reliability, the questionnaire includes five quasi-identical items. The items are arranged in a randomized block design to prevent adjacent placement of similar content. Each item’s dipoles are juxtaposed, with half of the items in each (sub)dimension presenting the statement indicating a deviation from the norm on the left, and the other half on the right.

The following provides descriptions of the individual (sub)dimensions as detailed by Pekala (1991, 30: 130 ff.):

The positive affect dimension consists of three subdimensions. Whereas the joy subdimen- sion assesses feelings of ecstasy and extreme happiness, the sexual excitement subdimension addresses the extent of “intense sexual feelings”. The love subdimension asks about feelings of love and loving-kindness.

The negative affect dimension monitors anger, sadness, and fear. The anger subdimension assesses feelings of being “very angry and upset” or enraged, while the sadness subdimension monitors feeling very, very sad or unhappy. The fear subdimension asks about feeling “very frightened” or being scared or afraid.

The altered experience dimension is composed of four subdimensions: altered body image, altered time sense, altered perception, and altered or unusual meaning. Altered body image addresses the extent to which subjects feel their bodily feelings expand into the world around them. Altered time sense addresses the extent to which “the flow of time changed drastically” or whether it seemed to “speed up or slow down”. Concerning perception, this subdimension assesses changes in the perception of the world in terms of color, form, size, shape, or perspective.

The imagery dimension is composed of two subdimensions: amount of imagery and vividness of imagery. Whereas imagery amount assesses the amount of imagery, the vividness subdimension assesses the extent to which visual imagery is “vivid and threee-dimensional” or “as clear and vivid as objects in the real world”.

[The] Attention [dimension] likewise is composed of two subdimensions: direction of attention and absorption. Whereas direction addresses whether attention is directed toward “internal subjective experience” or “toward the environment around me”. Absorption assesses whether the person was absorbed in what they were experiencing versus being “continually distracted by extraneous impressions”.

The self awareness dimension of the PCI assesses the extent to which the person is “aware of being aware of myself”, versus having “lost consciousness” of themselves or not being aware of oneself. The altered state of awareness dimension, on the other hand, assesses being in “an extraordinary unusual and nonordinary state of awareness” versus one ´s state of consciousness being no different than usual.

…The internal dialog [dimension] is addressed to the extent to which someone was silently talking to himself a great deal.

…The rationality dimension addresses whether thinking is clear and distinct, or rational and easy to comprehend, versus thinking being “confused and muddled” or “nonrational and very hard to comprehend”.

[The] volitional control [dimension] assesses the extent to which one has “complete control over what one is paying attention to” or is “willfully controlling” experience versus becoming passive and receptive to experience or having “images and thoughts pop into my mind without my control”.

[The] memory [dimension] assesses the subjects’ perceptions that they can remember just about everything that they experienced versus not being able to remember what they experienced.

…The arousal dimension is really a measure of the extent of muscular tension, that is, the extent to which the muscles of the body are “very tense and tight” versus not feeling “tension or tightness at all”.

There are specific reasons for selecting the PCI for our study. Firstly, the PCI includes subscales (Visual Imagery, Volitional Control) that enable the identification of mental imagery as dream-like. Secondly, the PCI has been widely used in various studies on altered states of consciousness, facilitating comparisons with other research findings. Thirdly, the items related to imaginative experiences in the PCI are easily comprehensible. Moreover, the PCI is considered an ideal tool for the primary assessment of changes in consciousness resulting from flotation-REST when compared to a baseline, as indicated by Johanson et al. (2011).

### Definitions of mythic ontology (DMO)

The concept of a “mythic” experience refers to cognitive patterns that predate the rise of modern scientific thought. In contrast to contemporary cognition, which has been shaped significantly by the Industrial Revolution and the Enlightenment, earlier cognitive modes frequently emphasized symbolic meaning and experiential depth over logical coherence. Historical evidence suggests that these alternative cognitive frameworks were prevalent throughout much of human history (Tedlock, 2005; Clottes and Lewis-Williams, 1998). Extensive research in fields such as cultural anthropology (Eliade, 1968), structuralism (Descola, 2011; Lévi-Strauss, 1968), and the philosophy of religion (Otto, 1917) has investigated these premodern cognitive modes.

Rather than viewing these earlier cognitive modes as primitive, we suggest that they offer a valid comparison point for understanding dream-like states, which often deviate from the linear logic of waking consciousness. It should be noted that the mythic worldview of premodern societies is not assumed to have impaired their ability to accurately distinguish between waking and dream-like states. As Lévy-Bruhl (1921) noted, such cognitive patterns reflect enduring aspects of human experience rather than a mere historical phase.

In contrast to the structured worldview of modernity, earlier cognitive frameworks perceived reality as intricately intertwined with symbolic or “numinous” entities. These entities, as described by Burkert (1977) and Eliade (1968), are not confined to specific locations or objects but manifest through both material and ideational forms. This mythic perspective views everything as imbued with divine beings or powers (Diels, 1951: 79), which are not limited by spatial or logical constraints but are encountered in all things and events within their sphere of influence. According to Schadewaldt (1978: 92), these numinous entities are better understood as forces rather than deities, acting both in nature and within the human psyche as real influences capable of affecting and shaping our experiences.

Philosophers such as Cassirer (1964) provided a foundational understanding of this non-modern cognition, viewing it as an autonomous system of thought. Hübner (1985) later refined these ideas by offering detailed definitions of space, time, and substance within this framework. For the purposes of our study, these definitions—referred to as “mythic ontology”—have been adapted to examine experiences in altered states of consciousness.

Tables 1–3 summarize Hübner’s definitions of space, time, and substance within non-linear cognitive frameworks. To assess the relevance of these definitions to participants’ experiences, we employed a visual analog scale ranging from 0 to 100%. Participants rated their experiences according to these criteria, providing empirical data on how these alternative cognitive structures align with their subjective experiences in sensory deprivation-induced altered states.

**Table 1:** Definitions of space in the mythic worldview, according to Hübner (1985).

No.	Definitions of mythic space
1	Mythic space is not a general medium in which objects are merely situated; rather, space and content form an inseparable unity.
2	Mythic space is discrete, consisting of isolated spatial elements that align to form the overall structure of space.
3	It is inhomogeneous because places within it have both relative and absolute positions (e.g., top, bottom) relative to each other.
4	It is anisotropic, since the direction in which an event sequence propagates is significant.
5	Not all locations can be situated within ordinary space; some are singularities (mythic places like Olympus, Tartaros, etc.).
6	Space is not defined metrically, meaning that objects within it do not necessarily have a measurable extent in three dimensions.

**Table 2:** Definitions of time in the mythic worldview, according to Hübner (1985).

No.	Definitions of mythic time
1	Mythic time is not a general medium in which events occur; rather, time and content form an inseparable unity.
2	Mythic objects are not situated at specific points within this temporal medium, as they cannot be marked within it; rather, they only manifest a particular sequence of events internally.
3	Mythic time is cyclical and unidirectional (e.g., like the seasons) and consists of distinct, partially independent temporal forms known as Archái (origin stories).

**Table 3:** Definitions of substance in the mythic worldview, according to Hübner (1985).

No.	Definitions of mythic substance
1	Mythic substance embodies the presence of a numinous individual. Consequently, it encompasses both ideational (mental) and material properties.
2	Numinous individuals are consistently present wherever the associated natural, social, or psychological processes take place.
3	Substances with psychological effects always manifest in physical locations, making them perceivable (e.g., in the diaphragm or chest).

Given the abstract nature of the ontological definitions, they were reworded to make them more accessible to participants while preserving their core theoretical meaning. For example, *mythic space No. 2*, which describes mythic space as consisting of isolated elements, was rephrased as: “My experience was not a continuous whole but consisted of independent places, each with its own theme.” Similarly, *mythic space No. 3*, which defines mythic space as inhomogeneous, was adapted to: “The places I experienced were not structured by natural laws but by their own forces and rules.” For *mythic time No. 2*, which posits that mythic objects are not tied to specific temporal points, the item was rephrased as: “My experiences cannot be assigned to any specific place or time in the real world, much like in fairy tales.” Finally, *mythic substance No. 3*, which describes mythic substance as manifesting physically, was expressed as: “I felt myself being infused and transformed by forces.” These reworded items maintain the essence of the original definitions while ensuring clarity and accessibility for lay participants.

### Procedure

Upon enrollment in the study, participants received comprehensive information about the study procedures. Prior to signing the Informed Consent form, they were given a tour of the laboratory and provided with information regarding flotation-REST and the concrete environmental circumstances they will be in during the experimental sessions. Demographic and medical data were collected. One to two hours in advance of the first Flotation-REST session, a baseline measurement was conducted using the PCI and the DMO.

Following the baseline measurement, participants underwent a 3-week flotation-REST program, consisting of four 90-min flotation-REST sessions. During the first session, participants were allowed to acclimate to the isolation environment, and no measurements were taken to account for the potential novelty effect. After sessions 2, 3, and 4 Participants had the opportunity to write down their imaginative experiences and completed the *PCI* questionnaire during the hour following each *Flotation-REST* session. The four sessions enabled participants to gain a well-informed understanding of the potential structures and underlying patterns in their dream-like experiences during flotation REST. Following the final session, they assessed these experiences using the DMO.

### Statistics

The participants’ state of consciousness, measured by the PCI, was the primary outcome, while the DMO served as the secondary outcome parameter.

The normal distribution of the sample was confirmed by the Kolmogorov–Smirnov test. Since specific differences needed to be identified through pairwise comparisons, significance analysis was conducted using independent *t*-tests. This method is appropriate for the small sample size and the study’s exploratory design, which aims to detect and characterize trends that may warrant further investigation in subsequent research.

For the evaluation of the PCI questionnaires, two-tailed *t*-tests for dependent samples were performed. The independent variable was the time of measurement (baseline, float-REST sessions 2, 3, and 4). The comparisons were made between the everyday waking state (baseline) and flotation-REST sessions 2, 3, and 4. The PCI dimensions and their subscales were used as the dependent variables. No alpha error correction was applied due to the explorative (not hypothesis-oriented) nature of the study. To ensure reliable data and avoid speculative responses, only the PCI results of participants with a score of 50% or higher on the “Memory” subscale were included for each respective session. This criterion was necessary because falling asleep during the hypnagogic state in flotation-REST is possible.

Similarly, DMO results were included only for participants who scored 50% or higher on the PCI “Memory” subscale in at least one flotation-REST session. For the inferential statistics of the DMO, the mean values of the dream-like experiences after the fourth flotation-REST session were compared to those of the everyday waking experience using a Mann–Whitney *U*-test. To address the hypothesis-driven multiple comparisons, *p*-values were corrected using the Holm-Bonferroni method. When calculating mean values on DMO’s visual analog scales (VAS; 0 to 100 mm), any mean falling within the 0 to 4 mm range is conservatively considered zero. This method accounts for potential inaccuracies in marking the zero point, which could otherwise lead to significant deviations from zero (Hawker et al., 2011; Jensen et al., 2003).

Effect sizes, specifically Cohen’s d (Cohen, 1988) with pooled standard deviation, were calculated to provide insights into the practical significance of statistically significant findings. Additionally, linear correlations were assessed using the Pearson correlation coefficient and interpreted following Cohen’s guidelines (1988).

## Results

### The state of consciousness during sensory deprivation

Out of the initial pool of 48 interested individuals, 37 met the study criteria. However, six participants did not complete the study, resulting in a final sample size of 31 individuals (6 men, 25 women) aged between 23 and 66 years (M = 32.08, SD = 10.15). Of the initial 31 participants, 29 achieved a score of 50% or higher on the PCI “Memory” subscale during the 2nd flotation-REST session, 26 during the 3rd session, and 21 during the 4th session (see *Statistics*). All participants managed to complete at least one flotation-REST session without falling asleep. None of the participants required the use of the isolation tank’s alarm button. The statistical tests revealed several significant differences between the everyday waking state (baseline) in the last 1–2 h prior to flotation-REST and the state experienced during flotation-REST. Tables 4, 5 demonstrate a significant increase from baseline during flotation-REST in the PCI dimensions of Altered State, Attention, Altered Experiences, Positive Affect, and Visual Imagery. Conversely, there was a significant decrease in the dimensions of Arousal, Negative Affect, and Rationality. The dimension Volitional Control consistently exhibited a highly significant reduction, aligning with the expected dream-like experiences during flotation-REST (see *Flotation-REST*). No significant changes were observed in the dimensions of Self-Awareness, Memory, and Internal Dialog, as well as in the sub-dimensions of Love and Fear.

**Table 4:** PCI results (means, standard deviations) of flotation-REST sessions compared to waking experience (baseline).

Dimensions Subdimensions	Baseline Mean (SD)	2. Float-REST Mean (SD)	3. Float-REST Mean (SD)	4. Float-REST Mean (SD)
Altered experience	0.94 (1.04)	2.72 (1.25) ***	3.20 (1.13) ***	3.49 (1.48) ***
Body image	1.11 (1.21)	3.56 (1.60) ***	3.74 (1.68) ***	3.67 (1.90) ***
Altered time sense	0.82 (1.23)	3.07 (1.70) ***	3.73 (1.56) ***	3.62 (1.92) ***
Altered perception	0.67 (1.02)	2.59 (1.42) ***	2.92 (1.39) ***	3.76 (1.73) ***
Altered meaning	1.10 (1.10)	1.93 (1.86)	2.46 (1.36) ***	3.05 (1.91) ***
Positive affect	2.45 (1.28)	2.42 (1.65)	2.35 (1.50)	2.94 (1.32)
Joy	2.05 (1.28)	2.47 (2.13)	2.88 (1.97)	3.38 (1.76) **
Sexual excitement	2.36 (1.85)	2.29 (2.29)	1.02 (1.73) *	2.29 (2.18)
Love	3.09 (1.44)	2.54 (1.73)	3.04 (1.94)	3.23 (1.93)
Negative affect	1.52 (1.35)	1.60 (1.46)	0.62 (0.98) *	0.75 (0.91) *
Anger	1.89 (1.65)	1.26 (1.50)	0.37 (0.94) ***	0.90 (1.48) *
Sadness	1.59 (1.58)	1.57 (1.73)	0.98 (1.47)	0.64 (1.11) *
Fear	1.25 (1.48)	1.87 (2.01)	0.58 (1.22)	0.69 (1.17)
Attention	2.47 (1.27)	3.93 (1.03) ***	4.04 (1.23) ***	3.98 (1.05) ***
Direction (inwards)	2.30 (1.39)	4.41 (1.07) ***	4.28 (1.18) ***	4.08 (1.16) ***
Absorption	2.78 (1.72)	3.21 (1.63)	3.67 (1.77)	3.83 (1.40) *
Visual imagery	3.07 (1.55)	4.12 (1.40) *	4.34 (1.19) **	4.71 (1.11) ***
Amount	2.74 (1.89)	4.38 (1.54) **	4.46 (1.36) **	4.83 (1.17) ***
Vividness	3.49 (1.28)	3.88 (1.41)	4.21 (1.27)	4.60 (1.18) **
Self-awareness	4.75 (1.51)	4.62 (1.10)	4.21 (1.34)	4.54 (1.03)
Altered state	0.98 (1.35)	3.28 (1.55) ***	4.08 (1.41) ***	4.17 (1.66) ***
Arousal	2.51 (1.71)	2.14 (1.71)	1.46 (1.77) *	1.71 (1.53)
Rationality	4.41 (1.54)	3.82 (1.22)	3.47 (1.54) *	4.11 (1.02)
Volitional control	4.16 (1.62)	2.61 (1.36) ***	2.01 (1.05) ***	2.22 (0.93) ***
Memory	4.50 (1.49)	4.66 (0.91)	4.45 (1.15)	4.78 (0.92)
Internal dialog	2.23 (2.09)	2.98 (1.98)	2.31 (2.03)	2.29 (2.21)
Reliability index score	1.28 (2.18)	0.89 (0.50)	0.57 (0.39)	0.52 (0.46)

Significances: * < 0.05; ** < 0.01; *** < 0.001.

**Table 5:** PCI results (*t*-values, degrees of freedom) of flotation-REST sessions compared to waking experience (baseline).

Dimensions Subdimensions	2. Float-REST Session *t*-value (df)	3. Float-REST Session *t*-value (df)	4. Float-REST Session *t*-value (df)
Altered experience	5.41 (49) ***	7.16 (46) ***	6.56 (41) ***
Body Image	5.99 (49) ***	6.12 (46) ***	5.29 (41) ***
Altered time sense	5.25 (49) ***	7.08 (46) ***	6.72 (41) ***
Altered perception	5.37 (49) ***	6.29 (46) ***	7.17 (41) ***
Altered meaning	1.86 (49)	3.74 (45) ***	4.13 (41) ***
Positive affect	0.07 (47)	0,24 (45)	1.22 (40)
Joy	0.82 (49)	1.69 (46)	2.84 (41) **
Sexual excitement	0,12 (49)	2.59 (46) *	0.11 (41)
Love	1.19 (47)	0.09 (45)	0.27 (40)
Negative affect	0.19 (47)	2.64 (45) *	2.18 (41) *
Anger	1.42 (49)	3.99 (46) ***	2.07 (41) *
Sadness	0.04 (48)	1,38 (46)	2.27 (41) *
Fear	1.20 (47)	1.70 (45)	1.37 (41)
Attention	4.53 (49) ***	4.34 (46) ***	4.24 (41) ***
Direction (inwards)	6.13 (49) ***	5.34 (46) ***	4.55 (41) ***
Absorption	0.91 (49)	1.76 (46)	2.19 (41) *
Visual imagery	2.51 (48) *	3.21 (46) **	3.97 (41) ***
Amount	3.38 (48) **	3.66 (46) **	4.34 (41) ***
Vividness	1.02 (49)	1.95 (46)	2.95 (41) **
Self-awareness	0.36 (49)	1.31 (46)	0.53 (41)
Altered state	5.54 (49) ***	7.74 (46) ***	6.93 (41) ***
Arousal	0.77 (49)	2.08 (46) *	1.61 (41)
Rationality	1.53 (49)	2.11 (46) *	0.75 (41)
Volitional control	3.71 (49) ***	5.54 (46) ***	4.79 (41) ***
Memory	0.47 (49)	0.13 (46)	0.74 (41)
Internal dialog	1.31 (49)	0.13 (46)	0.09 (41)
Reliability index score	0.92 (48)	1.63 (46)	1.56 (41)

Significances: * < 0.05; ** < 0.01; *** < 0.001.

### The phenomenology of dream-like experiences

The participants wrote down their imaginative experiences immediately after the flotation-REST session. Four exemplary excerpts are presented below to provide insight into the content, variations, and progression of the imaginations they experienced. Due to the high degree of imaginative autonomy, individuals often find themselves immersed in a narrative where they alternately experience the roles of both observer and active participant. The rapid scene changes are accompanied by role shifts, which are further linked to changes in perspective, as well as physical and psychological sensations.

*Excerpt 1*: “At first, I feel my body intensely and remain very conscious. Thoughts and atmospheric/physical images and sensations come and go (I feel like a lizard; it’s like being inside a giant ladybug, and so on). Memory images also emerge. Then, an image appears (a painting I like), and I step into the image, trying to sense and look around, which works well. A being (a woman) appears, and I make contact with her. The situation is very touching, and I linger in this image/scene for a while. Later, triggered by bodily sensations, another image appears. In it, I become a ‘fairy tale figure’ and move through a kind of fairy tale world. A few stories develop, and everything becomes very imaginative. Then the figure from the first image reappears and gives me a gift. Very empowering.”

*Excerpt 2*: “A hot air balloon floats through a mountainous landscape. Then I become the balloon myself and can smell the gas. The balloon changes colors, turning into a red balloon attached to a dog collar. In the new scene, I become the young dog wearing the collar. Then an image appears of a young woman driving a blue convertible through a mountain pass in South Tyrol. I slip into the role of the woman, feeling the temperature changes during the drive, but especially sensing her thoughts and emotions. Next, there’s a time jump. She now has a family, and the blue convertible sits dusty in the garage. The house is bright and welcoming; she has two daughters, and they are planning their vacation. Afterward, a bird scene unfolds: in rapid succession, I slip into the body of several bird species—blackbird, eagle, parrot, sparrow, and hummingbird. In the next scene, a young student is shown. She has black hair and green eyes and is preparing for a meeting with two of her friends. A 30-year-old attractive man with dark brown hair and blue eyes appears. They meet, have a lively and carefree conversation, and take a taxi together. At the apartment, they engage in sexual intercourse. In this scene, I alternately experience the role of both the woman and the man.”

*Excerpt 3*: “I am lying next to a bear; it is brown and warm. I feel its fur on my skin. We are in a cave, waking up together and moving toward the light. Then I relive the moment when my young friend Ruth was diagnosed with ‘inoperable brain tumor.’ I see her eyes, our despair, the embrace. Then I am back floating in the tank, but a light penetrates me, radiating from my center. Warmth accompanies it, and I feel the grief as a weight above my head. I let it flow through me down to my feet, like a trembling sensation. I feel like a burning flame, white and open. Then it passes, and I am myself again, sensing the heaviness in my heart. But something is better—there is a shift. I am closer to myself, more distinctly aware of my own being.”

*Excerpt 4*: “A snake is large and approaches me with its mouth open. It ‘eats’ me. Actually, it swallows me whole. I am inside it. Through its eyes, I can perceive the forest floor. I see earth, wood, grass, branches, insects. I smell the forest floor—damp, cozy, warming. The old skin itches; through the friction, the snake sheds it. I am the snake. I feel it and at the same time, I experience what it allows me to feel. The new skin is very sensitive and shimmers in many beautiful colors. It’s both pleasurable and exhausting. My pelvis moves my legs, and they swing back and forth in the tank. I am the snake. I marvel, and my mouth opens. I experience a deep sense of awe, respect, humility, and connection that is nearly beyond comparison. I am also two (or more) levels of consciousness at once. The imaginations merge with my reality, sending impulses that actually manifest in my body. An incomparable feeling of grandeur, love, and oneness!”

The participants’ experiences reveal characteristics that are consistent with the DMO framework, particularly the notion that each experienced narrative unfolds its own “world,” where the laws governing that world depend on its theme. The dynamic forces at play not only shape the world but also transform the participant’s perception and understanding of their own existence (e.g., by becoming a snake). In these narratives, the mythic logic operates through a transformation that is as much internal as external, blending the world of the story with the inner world of the perceiver.

### The ontology of dream-like experiences

Thirty-one participants assessed the DMO. Descriptive statistics indicate that all items received scores higher than 50 (on a scale from 0 to 100), indicating a closer proximity to the mythic pole for the experienced dream-like content. In contrast, all items scored below 50 for everyday waking experiences (baseline). The analyses revealed significant differences between dream-like and everyday waking experiences for all dimensions, thereby confirming the hypothesis. Refer to Table 6 for detailed results.

**Table 6:** Comparison of substance, time and space between everyday waking and dream-like experiences.

Dimension	Waking state Mean (SD) Median	Dream-like state Mean (SD) Median	*U*	*z*	*p*	*d*
Substance	24.88 (29.74) 8.5	56.39 (34.61) 62	1957	6.15	<0.001 ***	0.98
Time	17.46 (20.64) 8	68.95 (28.05) 79	741.5	9.76	<0.001 ***	2.09
Space	18.45 (24.94) 5.5	69.25 (30.48) 77.85	3,737.5	12.12	<0.001 ***	1.82

Significances: * < 0.05; ** < 0.01; *** < 0.001.

Furthermore, a *post hoc* analysis was conducted to examine the similarity between the everyday waking state of our participants and the modern pole. Significant disparities were found between the waking state of our participants and the ideal/pure modern worldview. To conduct this analysis, a Mann–Whitney *U*-Test was performed, comparing the mean values of the items with a value of 4 mm (instead of 0 mm). The selection of 4 mm was aimed at excluding potentially inaccurate markings on the visual analog scales that might occur within the range of 0 to 4 mm (see *Statistics*). Refer to Table 7 for detailed results.

**Table 7:** Comparison of substance, time and space between everyday waking experiences and the modern world model (value of 4 mm).

Dimension	Modern Worldview Median	Waking state M (SD/Median)	*W*	*z*	*p*
Substance	4	24.88 (29.74) 8.5	3.072	4.64	<0.001 ***
Time	4	17.46 (20.64) 8	3,398.5	5.17	<0.001 ***
Space	4	18.45 (24.94) 5.5	10.705	5.5	<0.001 ***

Significances: * < 0.05; ** < 0.01; *** < 0.001.

## Conclusion

This study clarifies the structural parallels between dream-like experiences induced by flotation-REST and premodern mythic cognition. Significant changes in the Phenomenology of Consciousness Inventory (PCI) dimensions—such as heightened altered states, diminished volitional control, increased focus on internal experiences, and more vivid visual imagery—contrast sharply with normative waking states. These findings align with Hruby et al. (2024) and suggest that flotation-REST experiences possess unique cognitive and perceptual characteristics similar to those found in dream-like and psychedelic states.

Our results indicate that the illogical and surreal aspects of these induced experiences exhibit substantial similarities with mythic thinking, a distinct cognitive mode grounded in a well-defined mythic ontology. This ontology diverges fundamentally from the scientific ontology that dominates modern cognition. Analysis reveals that these experiences exhibit ontological parallels with mythic conceptions of space, time, and substance (as outlined in Tables 1–3). Additionally, our finding that mythic cognition was partially present in participants’ waking states supports the contemporary view of consciousness as a continuum between waking and dreaming states (Kirberg, 2022; Christoff et al., 2016; Fox et al., 2013).

By using flotation-REST to elicit these experiences, we demonstrate that their perceived illogicality reflects a distinct ontological framework rather than cognitive deficits. The data support the hypothesis that these experiences align more closely with mythic structures than with modern, rationalistic perspectives. This alignment reveals a fundamental divergence from modern waking cognition, suggesting that dream-like states may be linked to premodern cognitive schemas.

While mythic cognition might be considered “irrational” from a modern perspective, contemporary theories, such as those by Varela et al. (1991), propose that cognition encompasses not only abstract reasoning but also embodied experience and meaning-making processes. Research on dream-like states, daydreams, and altered states of consciousness suggests that the brain’s default mode network (DMN), active during non-task-oriented thinking, generates experiences that are more associative and less bound by linear logic (Carhart-Harris et al., 2014). This suggests a spectrum of cognitive modes, integrating both modern rational thought and symbolic, associative processes akin to mythic cognition.

In conclusion, dream-like states appear to arise from non-modern ways of experiencing and interpreting reality rather than from cognitive deficits. This broader perspective enhances our understanding of consciousness and underscores the value of incorporating mythic perspectives into modern cognitive research. The findings of this study support the view that mythic cognition should not be dismissed as a relic of archaic thought but rather recognized as an integral dimension of human experience. The perception of this cognitive dynamic in flotation REST-induced states parallels recent approaches in cognitive neuroscience, which emphasize the importance of associative and non-linear thought processes (cf. Carhart-Harris et al., 2014). Such perspectives may provide new impetus for transdisciplinary research, viewing mythical and scientific worldviews not as oppositional but as complementary. For future studies, we recommend developing and validating new questionnaires to capture the nuanced aspects of mythic ontologies, drawing on scientific research into premodern cognitive frameworks. Additionally, further theoretical research is suggested to explore how our findings align with contemporary psychological theories and philosophical perspectives.

## Limitations

This exploratory study offers important insights into the connection between dream-like states and mythic cognition, consistent with existing research on sensory isolation. However, notable limitations must be addressed. The absence of a control group in the within-subject design and the small sample size of 31 participants restrict the generalizability and robustness of the findings. Additionally, while the study supports the continuum between mythic and ordinary waking consciousness, further validation of the questionnaire items is needed. Future research should focus on refining and validating these items to ensure they reliably distinguish different states of consciousness. The study’s exploratory nature reveals significant trends but necessitates validation through larger and more diverse samples. Despite these limitations, the findings contribute to the understanding of dream-like experiences and mythic cognition, underscoring the need for further research to confirm and extend these results.

## Data Availability

The raw data supporting the conclusions of this article will be made available by the authors, without undue reservation.
